# Draft Genome Sequence of a Methicillin-Resistant *Staphylococcus aureus* Isolate (Sequence Type 1) from Seafood

**DOI:** 10.1128/genomeA.00776-17

**Published:** 2017-08-24

**Authors:** G. K. Sivaraman, Deesha Vanik, S. Visnuvinayagam, M. M. Prasad, C. N. Ravishankar

**Affiliations:** a Regional Centre of ICAR-Central Institute of Fisheries Technology, Veraval, Gujarat, India; b Regional Centre of ICAR-Central Institute of Fisheries Technology, Vashi, Navi Mumbai, Maharashtra, India; c ICAR-Central Institute of Fisheries Technology, Willingdon Island, Cochin, Kerala, India

## Abstract

The draft genome sequence of a methicillin-resistant *Staphylococcus aureus* (MRSA) isolate (sequence type 1 [ST 1]) from the salted dried ribbonfish from Gujarat, India, is reported here. *Staphylococcus* genus-specific genes were present in this MRSA isolate. The whole-genome sequence of this strain contains 2,797 protein-coding genes and 80 RNAs within the 2.85-Mb genome.

## GENOME ANNOUNCEMENT

*Staphylococcus aureus* is a common inhabitant of human skin and noses of 25% of healthy people. It is a well-known opportunistic pathogen that can cause a broad range of infections from mild skin infection to invasive diseases (1–3). It is resistant to all beta-lactam antibiotics, methicillin, oxacillin, penicillin, and amoxicillin, except anti-methicillin-resistant *Staphylococcus aureus* (MRSA) cephalosporins (4). Coagulase-positive *Staphylococcus aureus* (MRSA) strains are internationally acknowledged as zoonotic multidrug-resistant pathogenic bacteria responsible for nosocomial and community-acquired infections (5). Multidrug-resistant *S. aureus* strains are rather common in hospitals and farms but are also detected in food animals, such as pork, beef, and chicken, and in milk and fishery products in Europe, the United States, and Asia (6). The incidence of MRSA in fish and seafood was recently noted (7, 8). Food contamination with antibiotic-resistant MRSA can be a major threat to public health, as this resistance can be transferred through resistant MRSA strains to human clinical significance (9).

The genome of the MRSA strain was sequenced commercially and assembled to determine the genetic structure of its multiple drug resistance, including methicillin resistance (*mecA* and *femA*). Fish samples were collected on tryptic soy broth (Oxoid, United Kingdom) and incubated at 35°C for 18 to 24 h, and a loopful of culture was streaked onto a MRSA II agar plate (Difco, USA). DNA was extracted from a typical mauve-colored colony employing a bacterial genomic DNA isolation kit (Sigma, France), and genomic DNA quality was checked on a NanoDrop spectrophotometer. The whole-genome sequencing was carried out in Illumina HiSeq 2500 (paired end). The number of paired-end reads was approximately 7 billion short-read sequences in pairs of ~300 bp, the number of bases (Mb) was 1,447.5, and there was 35.11% G+C content. *De novo* contig assembly was performed using MaSuRCA (10), and further downstream processing was performed. Coding sequences (CDSs) were predicted from the contigs using Glimmer (11), and 2,792 predicted CDSs were found. The predicted CDSs were annotated using the in-house pipeline CANoPI (Contig Annotator Pipeline) in comparison with the NCBI database using the BLASTX program. Organism annotation, gene and protein annotation to the matched genes, gene ontology annotation, and pathway annotation were carried out with the use of the NCBI database. The predicted CDSs were compared with the NCBI nonredundant protein database using the BLASTX program. Matches with an *E* value of ≤10^−5^ and similarity score of ≥50% were retained for further annotation. Overall, we observed that 2,757 (98.57%) of the predicted CDSs had at least one hit in the NCBI database. Nearly 86% of the CDSs found using BLASTX have a confidence level of at least 1 × 10^−5^, which indicates high protein conservation. We found that 100% of the predicted CDSs have a similarity of more than 60% at the protein level with the existing proteins at the NCBI database. The majority of the top BLASTX hits belong to *Staphylococcus* species (top 15 organisms). Among the total significant BLASTX hit CDSs, 1,741 genes were annotated using the UniProt database. The total number of Gene Ontology annotations identified for molecular functions was 870, with 586 annotations having to do with a biological process and 236 annotations having to do with cellular components. We predicted tRNA genes from the contigs using tRNAscan-SE (12) and found 80 genes.

### Accession number(s).

This whole-genome shotgun project has been deposited at NCBI GenBank under the accession number NBZX00000000.
